# Fetal cardiovascular magnetic resonance feature tracking myocardial strain analysis in congenital heart disease

**DOI:** 10.1016/j.jocmr.2024.101094

**Published:** 2024-09-13

**Authors:** Thomas M. Vollbrecht, Christopher Hart, Christoph Katemann, Alexander Isaak, Claus C. Pieper, Daniel Kuetting, Ulrike Attenberger, Annegret Geipel, Brigitte Strizek, Julian A. Luetkens

**Affiliations:** a Department of Diagnostic and Interventional Radiology, University Hospital Bonn, Bonn, Germany; b Quantitative Imaging Lab Bonn (QILaB), University Hospital Bonn, Bonn, Germany; c Department of Pediatric Cardiology, University Hospital Bonn, Bonn, Germany; d Philips GmbH Market DACH, Hamburg, Germany; e Department of Obstetrics and Prenatal Medicine, University Hospital Bonn, Bonn, Germany

**Keywords:** Fetal CMR, Doppler US, Feature tracking, Longitudinal strain, Congenital heart disease

## Abstract

**Background:**

Cardiovascular magnetic resonance (CMR) is an emerging imaging modality for assessing the anatomy and function of the fetal heart in congenital heart disease (CHD). This study aimed to evaluate myocardial strain using fetal CMR feature tracking (FT) in different subtypes of CHD.

**Methods:**

Fetal CMR FT analysis was retrospectively performed on four-chamber cine images acquired with Doppler ultrasound gating at 3T. Left ventricular (LV) global longitudinal strain (GLS), LV global radial strain (GRS), LV global longitudinal systolic strain rate, and right ventricular (RV) GLS were quantified using dedicated software optimized for fetal strain analysis. Analysis was performed in normal fetuses and different CHD subtypes (d-transposition of the great arteries [dTGA], hypoplastic left heart syndrome [HLHS], coarctation of the aorta [CoA], tetralogy of Fallot [TOF], RV-dominant atrioventricular septal defect [AVSD], and critical pulmonary stenosis or atresia [PS/PA]). Analysis of variance with Tukey post-hoc test was used for group comparisons.

**Results:**

A total of 60 fetuses were analyzed (8/60 (13%) without CHD, 52/60 (87%) with CHD). Myocardial strain was successfully assessed in 113/120 ventricles (94%). Compared to controls, LV GLS was significantly reduced in fetuses with HLHS (−18.6±2.7% vs −6.2±5.6%; p<0.001) and RV-dominant AVSD (−18.6±2.7% vs −7.7±5.0%; p = 0.003) and higher in fetuses with CoA (−18.6±2.7% vs −25.0±4.3%; p = 0.038). LV GRS was significantly reduced in fetuses with HLHS (25.7±7.5% vs 11.4±9.7%; p = 0.024). Compared to controls, RV GRS was significantly reduced in fetuses with PS/PA (−16.1±2.8% vs −8.3±4.2%; p = 0.007). Across all strain parameters, no significant differences were present between controls and fetuses diagnosed with dTGA and TOF.

**Conclusion:**

Fetal myocardial strain assessment with CMR FT in CHD is feasible. Distinct differences are present between various types of CHD, suggesting potential implications for clinical decision-making and prognostication in fetal CHD.

## Graphical abstract

**Figure:**
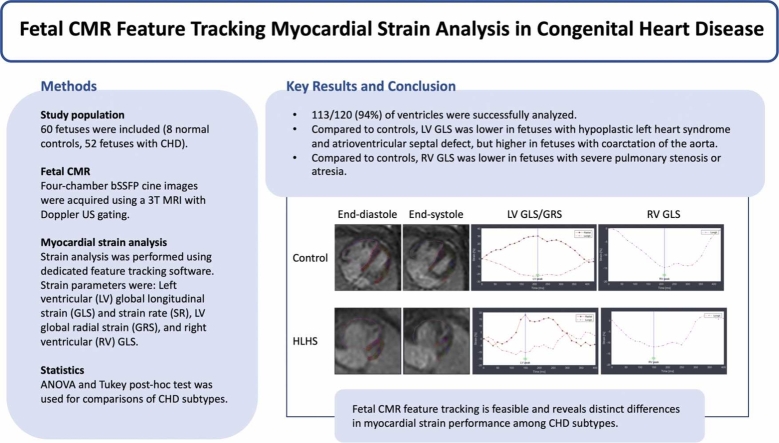

## 1 Introduction

Congenital heart disease (CHD) is the leading cause of major congenital anomalies, accounting for nearly one-third of all cases, with a reported prevalence ranging between 4 and 10 per 1000 live births [1], [2]. Depending on the underlying pathology, prompt pharmaceutical or surgical intervention may be imperative following birth, often accompanied by long-term medical management [3]. For evidence-based parental counseling and to gauge the presence of cardiovascular compromise, which impacts fetal and postnatal prognosis, it is decisive to be able to repetitively evaluate cardiac function throughout fetal development until birth [4], [5]. Until recently, due to the absence of alternatives, fetal ultrasound (US) has been the sole clinically utilized imaging modality for prenatal assessment of CHD. This involves second-trimester screening US and subsequent dedicated fetal echocardiography for precise diagnostic evaluation and ongoing monitoring in the event of detected anomalies [6]. In addition to cardiovascular morphology assessment, quantitative parameters can be measured to evaluate fetal cardiac function, such as quantifying ventricular wall deformity as an indicator of myocardial contractility [7]. In fetal CHD, deformation parameters, such as longitudinal strain and strain rate (SR), have been observed to differ from those in normal fetuses, reflecting distinct cardiac loading conditions [8]. As myocardial functional analysis might emerge as a quantitative determinant in determining the optimal delivery time for a compromised fetus [9], it is essential that assessment remains feasible even during advanced third-trimester pregnancy. Nonetheless, fetal echocardiography may be limited, particularly in advanced gestation, due to diminishing amniotic fluid volume, increasing bone calcification, or adverse fetal position. The introduction of alternative gating methods to replace the postnatal electrocardiogram (ECG), such as Doppler US (DUS) gating, has made it possible to leverage the benefits of cardiovascular magnetic resonance (CMR) as a valuable adjunct imaging modality to echocardiography during prenatal life [10]. For example, it has been demonstrated that fetal CMR is capable of reliably assessing complex forms of CHD, even identifying additional pathologic findings that elude detection by echocardiography [11], [12]. Moreover, additional fetal CMR scans have been shown to aid clinical decision-making when echocardiography results are inconclusive [13]. Also, DUS-gated fetal CMR enables the assessment of cardiac function. Recently, reference values for various functional parameters in normal third-trimester fetuses, including left ventricular (LV) longitudinal strain, have been reported [14]. In addition, myocardial strain assessment utilizing CMR feature tracking (FT) has been demonstrated to be feasible in fetuses both with and without CHD [15]. However, the pathophysiological mechanisms leading to alterations in fetal hemodynamics and ventricular function vary widely depending on the underlying malformation. With this study, we aimed to evaluate fetal strain using CMR FT in different subtypes of CHD.

## 2 Material and methods

### 2.1 Study population

This retrospective analysis was conducted as part of a prospectively enrolled study cohort and approved by the institutional review board of the University Hospital Bonn. Pregnant women who were suspected of having a cardiovascular or other thoracic malformation of the fetus during second-trimester screening US scan were referred to the Department of Obstetrics and Prenatal Medicine at University Hospital Bonn for further diagnostic evaluation and pregnancy follow-up. Subsequently, they were invited to participate in this study and underwent fetal CMR scheduled from the 32nd week of pregnancy onward. Before fetal CMR, all participants provided written informed consent for participation and publication of images. Exclusion criteria included lack of consent to participate in the study and general magnetic resonance imaging (MRI) contraindications. Following birth, postnatal echocardiography and/or surgery reports were utilized to confirm prenatal diagnoses. Fetuses without cardiovascular malformations were classified as normal control group.

### 2.2 Fetal CMR acquisition

All fetuses underwent examination using a whole-body 3T MRI system (Ingenia Elition X, Philips Healthcare, Best, The Netherlands). Signal reception was facilitated by an anterior 16-channel torso coil and a posterior coil integrated into the patient table. The specific absorption rate was set to normal mode with a maximum limit of 2.0 W/kg. For retrospective gating of CMR sequences, an MRI conditional Doppler US gating device was employed (smart-sync, Northh Medical GmbH, Hamburg, Germany). In brief, the US transducer detects the Doppler signal of the fetal heartbeat and converts it into a gating signal, which is wirelessly transmitted to the ECG unit of the MRI system for synchronization of image acquisition. Pregnant women were typically scanned in the left decubitus position to prevent compression of the vena cava inferior and optimize comfort; however, the option of the supine position was also offered according to their preference. First, an MRI survey was performed to plan the application of the DUS transducer, ensuring precise alignment of the acoustic beam with the fetal heart. Subsequently, the transducer was positioned on the maternal abdomen using headphones for an acoustic feedback of the fetal heart. Once a stable signal was obtained, the transducer was secured with an elastic belt.

The imaging protocol consisted of standard T2-weighted single-shot turbo spin echo images in both axial and sagittal planes oriented along the fetus to visualize extra-cardiac thoracic anatomy, including the lungs and thoracic vessels, and aid in further planning. Typical parameters for the axial sequence were as follows: field of view: 350 × 350 mm^2^; acquisition matrix: 292 × 251 mm; repetition time: 2630 ms; echo time: 80 ms; flip angle: 90°; echo train length: 105; slices: 15; slice thickness: 4 mm; interslice gap: −1 mm; sensitivity encoding factor: 2; acquisition time: 39 s. For functional myocardial strain analysis, a segmented two-dimensional balanced steady-state free precession (bSSFP) cine sequence in a four-chamber view orientation was used, covering the fetal heart and supra-aortic branches as an axial continuous stack. Images were acquired during maternal end-expiratory breath-hold. Imaging parameters typically employed were as follows: field of view: 254 × 254 mm^2^; repetition time: 4.2 ms; echo time: 2.1 ms; shot duration/acquisition time: 25.5 ms; flip angle: 65°; in-plane resolution: 1.72 × 1.45 mm^2^ (reconstructed: 0.99 × 0.99 mm^2^); slices: 16; slice thickness: 4 mm; no interslice gaps; average breath-hold duration/slice: 8 s; temporal resolution: 17.5 ms; reconstructed cardiac phases: 25; parallel imaging factor: 2.

### 2.3 Fetal CMR-derived feature tracking

Fetal LV and right ventricular (RV) strain parameters were quantified by utilizing CMR FT from four-chamber bSSFP cine images using a dedicated software (Segment, Version 2.1.R.6108, Medviso, Lund, Sweden). This software estimates myocardial strain curves by computing inter-frame deformation fields through a B-spline tensor product transform-based tracking strategy [16]. It employs an iterative process using intensity-based similarity metrics and regularization terms to ensure spatial smoothness of the deformation field. Unlike tracking myocardial boundaries only, it utilizes the entire image content for optimization and incorporates a temporal coherence strategy, enhancing robustness to image quality issues [16]. The software model used in this study was optimized by the vendor for fetal applications to accommodate high-resolution data and smaller imaging objects; however, the underlying tracking algorithm remained unchanged from the non-fetal version. Following the determination of the cardiac cycle by identifying end-diastole and end-systole images, the RV endocardial contour was manually delineated in the end-diastolic image, beginning at the edge of the tricuspid valve annulus, extending to the apex, and returning to the other previously defined edge. Similarly, the LV endo- and epicardial contours were manually traced using the same dataset. Papillary muscles were not included. Subsequently, RV and LV global longitudinal strain (GLS), LV global radial strain (GRS), and LV global longitudinal systolic SR were calculated by automatically propagating the manually drawn ventricular contours throughout the cardiac cycle and displayed as curves. In cases where automatic tracking was deemed inadequate upon visual inspection, contour delineation was repeated and re-propagated. If contours could not be successfully propagated after more than 10 attempts, analysis of this ventricle was considered not applicable. Analysis was performed by T.M.V., a cardiovascular radiologist with 4 years of experience in CMR of CHD (reader 1). In a subset of 15 randomly selected exams, analysis was additionally performed by J.A.L., a board-certified cardiovascular radiologist with 12 years of experience in CMR of CHD (reader 2). After a washout period of 2 weeks, the same 15 fetal CMR scans were again analyzed by reader 1.

### 2.4 Statistical analysis

Prism (Version 9.5.1; GraphPad Software, San Diego, California) and SPSS (Version 27, IBM Corp., Armonk, New York) were used for statistical analysis. Dichotomous variables were summarized as percent of absolute frequency. Continuous variables were checked for Gaussian normal distribution using the Shapiro-Wilk test and reported as mean ± standard deviation. Student’s t test was used to determine differences between fetuses with CHD and controls. Group differences between different CHD subgroups and controls were assessed using one-way analysis of variance followed by Tukey multiple comparison tests. Inter- and intra-observer reproducibility of strain measurements in the 15 randomly selected exams were assessed by Bland-Altman analysis and intraclass correlation coefficients (ICC). ICC estimates and their 95% confidence interval were based on a single-measure, two-way mixed (consistency) model (<0.5: poor, 0.5–0.75: moderate; 0.75–0.9: good, >0.9: excellent). A p-value of <0.05 was considered to indicate a significant difference.

## 3 Results

**Fig. 1:**
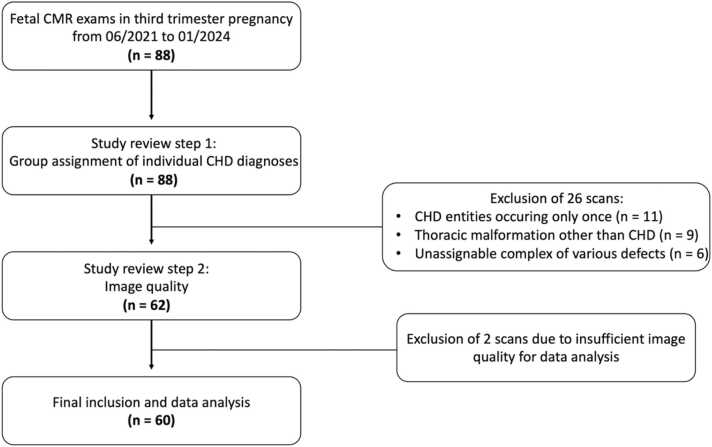
Study flowchart. *CHD* congenital heart disease, *CMR* cardiovascular magnetic resonance

A total of 88 consecutive fetal CMR exams, which were acquired between June 2021 and January 2024, were reviewed for potential inclusion in the study. Of these, 26 (30%) fetuses could not be assigned to a specific CHD type as previously defined and were thus excluded from further analysis (Fig. 1). From the remaining 62 fetal CMR studies considered for inclusion, 2 (3%) were excluded due to inadequate image quality. The final study collective consisted of 60 fetuses. Exactly 52/60 (87%) fetuses were diagnosed with a specific CHD type: d-transposition of the great arteries (dTGA; n = 10), hypoplastic left heart syndrome (HLHS; n = 13), coarctation of the aorta (CoA; n = 9), tetralogy of Fallot (TOF; n = 7), RV-dominant atrioventricular septal defect (AVSD; n = 7), and critical pulmonary stenosis or atresia (PS/PA; n = 6). Exactly 8/60 (13%) fetuses were classified as normal controls without cardiovascular anomalies, which was confirmed by postnatal echocardiography. Mean gestational age of all included 60 fetuses at the time of CMR was 249 ± 8.5 days (range: 226–266 days), without difference between control fetuses and fetuses with CHD (249 ± 6.7 days vs 248 ± 8.8 days; p = 0.780). Clinical characteristics of the study population are detailed in Table 1.

**Table 1:** Clinical characteristics of the study population.

	All patients n = 60	CHD n = 52	Controls n = 8	p-value
Maternal age (y)	32.6 ± 4.7	32.2 ± 4.6	35.8 ± 4.9	**0.044**
Maternal weight (kg)	81.0 ± 19.5	81.1 ± 20.6	80.5 ± 10.7	0.940
Maternal BMI (kg/m^2^)	29.7 ± 6.4	30.0 ± 6.7	28.1 ± 4.0	0.456
Gestational age (days)	249 ± 8.5	248 ± 8.8	249 ± 6.7	0.780
Fetal heart rate (bpm)	137 ± 19.8	138 ± 18.9	136 ± 9.6	0.759
CHD entity				
d-Transposition of the great arteries		10 (19)		
Hypoplastic left heart syndrome		13 (25)		
Coarctation of the aorta		9 (17)		
Tetralogy of Fallot		7 (13)		
Atrioventricular septal defect		7 (13		
Pulmonary valve stenosis/atresia		6 (13)		

Data are means ± standard deviation or numbers with percentages in parentheses. Bolded p-values indicate statistical significance.

*BMI body mass index, CHD congenital heart disease*

### 3.1 Feasibility of fetal myocardial strain analysis

Adequate tracking of ventricular borders throughout the cardiac cycle and assessment of all myocardial strain parameters was feasible in 113/120 (94%) ventricles. In 6/120 ventricles (5%), CMR FT could not be successfully conducted after more than 10 attempts due to the inability to automatically propagate the contours because of too small ventricular size (3 LV in fetuses with HLHS and 3 LV in fetuses with RV-dominant AVSD). Additionally, in 1/120 ventricles (0.8%), strain measurements were disregarded due to inadequate border tracking (1 LV in a fetus with PS/PA).

### 3.2 Comparison of strain parameters between controls and fetuses with CHD

No significant differences were present in strain parameters between normal control fetuses and pooled fetuses with CHD (Table 2). When comparing individual subgroups, significant differences were present in all strain parameters, indicating a wide range of values across the different CHD entities: The lowest LV GLS were observed in HLHS fetuses with mitral and aortic valve atresia, while the highest were found in fetuses with CoA (−6.2 ± 5.6% vs −25.0 ± 4.3%; p < 0.001). Similarly, RV GLS ranged from the lowest values in fetuses with PS/PA to the highest values in fetuses with CoA (−8.3 ± 4.2% vs −21 ± 3.3%; p < 0.001).

**Table 2:** Left ventricular (LV) and right ventricular (RV) systolic strain measurements in fetuses with congenital heart disease (CHD) and normal control fetuses.

	CHD n = 52	Controls n = 8	p-value
LV global longitudinal strain (%)	−15.6 ± 8.1	−18.6 ± 2.7	0.314
LV global radial strain (%)	19.8 ± 11.0	25.7 ± 7.5	0.154
LV strain rate (s^−1^)	−1.6 ± 0.6	−1.6 ± 0.3	0.908
RV global longitudinal strain (%)	−16.2 ± 5.1	−16.1 ± 2.8	0.943

Data are means ± standard deviation. *CHD* congenital heart disease, *LV* left ventricular, *RV* right ventricular

**Fig. 2:**
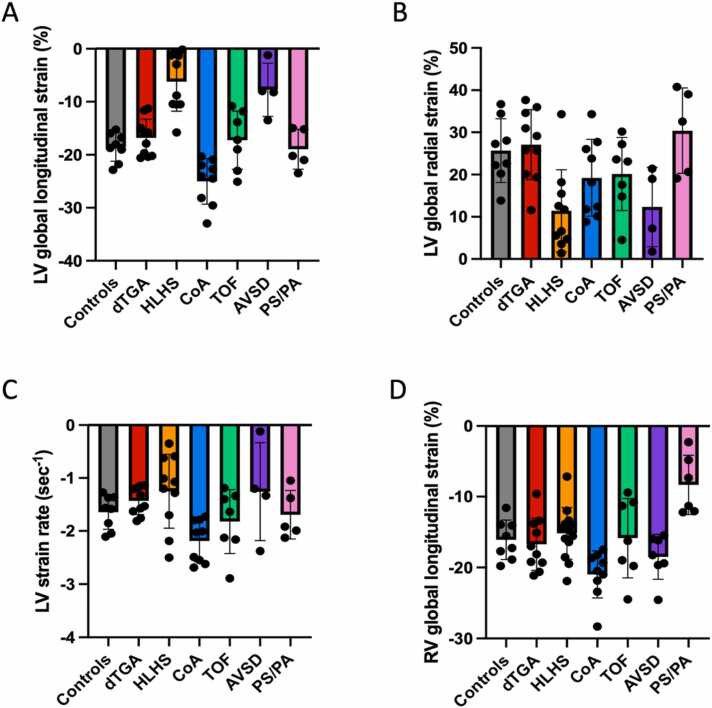
Bar plots showing strain measurements across different CHD subgroups and for normal control fetuses. *AVSD* atrioventricular septal defect, *CHD* congenital heart disease, *CoA* coarctation of the aorta, *dTGA* d-transposition of the great arteries, *HLHS* hypoplastic left heart syndrome, *LV* left ventricle, *PS/PA* pulmonary stenosis/atresia, *RV* right ventricle, *TOF* tetralogy of Fallot

**Fig. 3:**
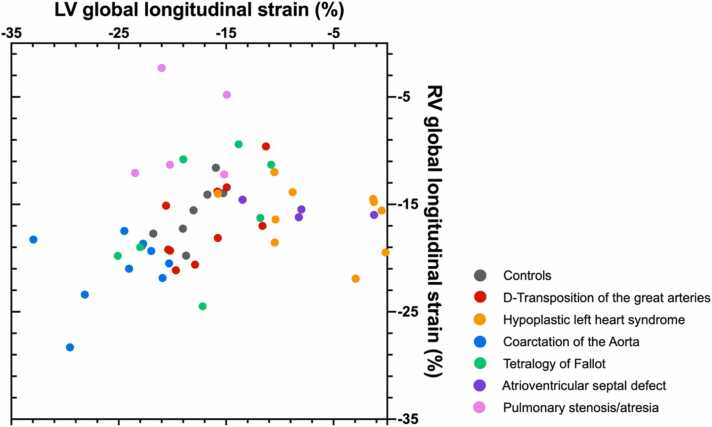
Scatter plot showing left ventricular (LV) and right ventricular (RV) global longitudinal strain measurements in individual fetuses with congenital heart disease and normal control fetuses. A wide range of LV and RV global longitudinal strain values were present across different CHD entities with considerable overlap with normal values particularly in fetuses with d-transposition of the great arteries and tetralogy of Fallot. *CHD* congenital heart disease

**Fig. 4:**
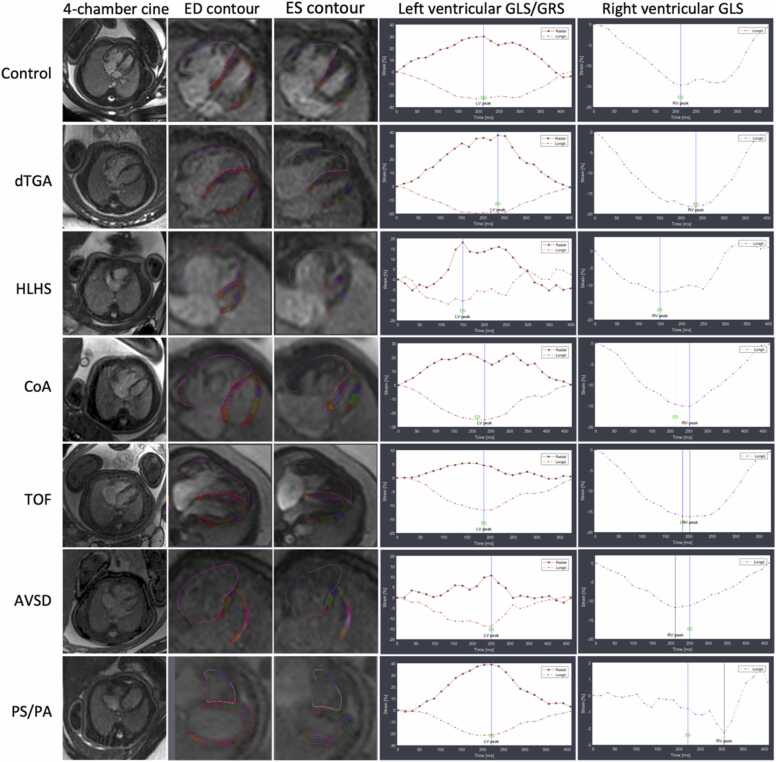
Left and right ventricular strain measurements displayed as curves across the cardiac cycle of different CHD entities including corresponding end-diastolic (ED) and end-systolic (ES) endocardial border contours. Please note that the hypoplastic left ventricle in the fetus with hypoplastic left heart syndrome (HLHS), the left ventricle in the fetus with right ventricular-dominant atrioventricular septal defect (AVSD), and the right ventricle in the fetus with pulmonary stenosis/atresia exhibit irregular deformity patterns over the cardiac cycle, diverging from the typical strain curves observed in the other fetuses. *CHD* congenital heart disease, *CoA* coarctation of the aorta, *dTGA* d-transposition of the great arteries, *GLS* global longitudinal strain, *GRS* global radial strain, *LV* left ventricular, *RV* right ventricular, *TOF* tetralogy of Fallot

Compared to normal control fetuses, LV GLS was lower in fetuses with HLHS (−18.6 ± 2.7% vs −6.2 ± 5.6%; p < 0.001) and RV-dominant AVSD (−18.6 ± 2.7% vs −7.7 ± 5.0%; p = 0.003) and higher in fetuses with CoA (−18.6 ± 2.7% vs −25.0 ± 4.3%; p = 0.038). Similar to normal control fetuses, these differences were also present when compared to fetuses with dTGA, TOF, and PS/PA. Compared to normal control fetuses, LV GRS was lower in fetuses with HLHS (25.7 ± 7.5% vs 11.4 ± 9.7%, p = 0.024). Compared to normal control fetuses and all other CHD subgroups, RV GRS was significantly lower in fetuses with critical PS/PA (−16.1 ± 2.8% vs −8.3 ± 4.2%, p = 0.007). Complete strain results are detailed in Table 3 and displayed in Fig. 2, Fig. 3, Fig. 4 .

**Table 3:** Left ventricular (LV) and right ventricular (RV) systolic strain measurements in fetuses with different forms of congenital heart disease and normal control fetuses.

	Controls	dTGA	HLHS	CoA	TOF	AVSD	PS/PA	p-value
LV GLS (%)	−18.6 ± 2.7^a^^,^^b^^,^^c^	−16.8 ± 3.5^a^^,^^c^	−6.2 ± 5.6^b^^,^^d^^,^^e^^,^^f^^,^^g^	−25.0 ± 4.3^a^^,^^c^^,^^d^^,^^e^^,^^f^	−17.2 ± 5.5^a^^,^^b^^,^^c^	−7.7 ± 5.0^b^^,^^d^^,^^e^^,^^f^^,^^g^	−19.0 ± 3.8^a^^,^^c^	<0.001
LV GRS (%)	25.7 ± 7.5^a^	27.1 ± 8.3^a^	11.4 ± 9.7^d^^,^^e^^,^^g^	19.2 ± 9.1	20.1 ± 8.7	12.3 ± 9.4	30.4 ± 10.1	<0.001
LV SR (s^−1^)	−1.6 ± 0.3	−1.4 ± 0.3^b^	−1.3 ± 0.7^b^	−2.2 ± 0.4^a^^,^^e^	−1.8 ± 0.6	−1.3 ± 0.9	−1.7 ± 0.5	0.008
RV GLS (%)	−16.1 ± 2.8^g^	−16.7 ± 3.7^g^	−15.2 ± 3.6^b^^,^^g^	−21.0 ± 3.3^a^^,^^g^	−15.9 ± 5.6^g^	−18.5 ± 3.2^g^	−8.3 ± 4.2^a^^,^^b^^,^^c^^,^^d^^,^^e^^,^^f^	<0.001

Data are means ± standard deviation. p-Values were obtained using one-way analysis of variance followed by Tukey multiple comparisons test

*AVSD* atrioventricular septal defect, *CoA* coarctation of the aorta, *dTGA* d-transposition of the great arteries, *GLS* global longitudinal strain, *GRS* global radial strain, *HLHS* hypoplastic left heart syndrome, *LV* left ventricle, *PS/PA* pulmonary stenosis/atresia, *RV* right ventricle, *SR* strain rate, *TOF* tetralogy of Fallot

a p < 0.05 compared to HLHS

b p < 0.05 compared to CoA

c p < 0.05 compared to AVSD

d p < 0.05 compared to controls

e p < 0.05 compared to dTGA

f p < 0.05 compared to TOF

g p < 0.05 compared to PS/PA

### 3.3 Intra- and inter-observer reproducibility

**Fig. 5:**
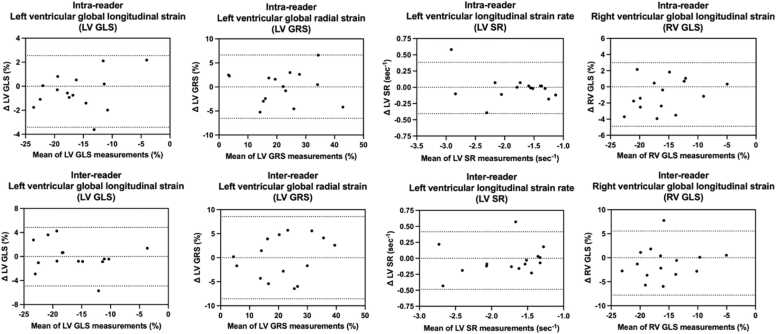
Bland-Altman plots for all myocardial strain parameters measured by readers 1 and 2.

Reproducibility across all strain parameters was good and excellent for both intra- and inter-observer measurements, with minimal disparity between the two readers. Bland-Altman analyses revealed minimal systematic biases in most measurement comparisons conducted by the two readers. ICC and reproducibility results are presented in Table 4 and Bland-Altman plots are given in Fig. 5.

**Table 4:** Intra- and inter-observer reproducibility for left ventricular (LV) and right ventricular (RV) strain measurements.

	Intra-observer reproducibility		Inter-observer reproducibility	
	ICC	95% CI	ICC	95% CI
LV global longitudinal strain	0.98	0.94–0.99	0.95	0.86–0.99
LV global radial strain	0.98	0.93–0.99	0.95	0.86–0.98
LV strain rate	0.97	0.90–0.99	0.95	0.84–0.98
RV global longitudinal strain	0.96	0.87–0.99	0.87	0.61–0.96

Data representing reproducibility assessments based on intraclass correlation coefficients (ICC) with corresponding 95 % confidence intervals (CI)*. LV* left ventricular, *RV* right ventricular

## 4 Discussion

This study presents LV and RV strain values in fetuses with various types of CHD utilizing DUS-gated fetal CMR FT. While strain measurements did not show significant differences between normal control fetuses and pooled fetuses with CHD, notable variations were observed across different CHD types, with significant differences between controls and fetuses with HLHS, CoA, RV-dominant AVSD, and critical PS/PA.

Since the introduction of myocardial tagging, strain assessment by CMR has been realized by various techniques, including FT [17], [18]. By tracking anatomical features alongside the myocardial boundaries over the cardiac cycle, accurate measurements of both ventricular and atrial strain can be provided [19]. Clinical ability of the method has been demonstrated in a wide variety of cardiovascular diseases, yielding excellent reproducibility [18]. Until recently, myocardial deformation analysis in prenatal life was only achievable through Doppler tissue imaging or, more recently, two-dimensional speckle tracking echocardiography (STE) [20]. Since the initial report of normal fetal deformation parameters in 1999 [21], myocardial strain imaging has been investigated in various conditions of altered hemodynamics resulting from both maternal and fetal disease, including CHD [22], [23], [24]. While speckle tracking may offer advantages over other US techniques by being less reliant on fetal position, adequate image quality is essential to avoid inaccuracies stemming from poor endocardial delineation [9], [25]. Particularly in the advanced third trimester, when substantial changes in ventricular preload and afterload affect myocardial strain (e.g., due to changing pressure gradient between the atria, increase in pulmonary blood flow and venous return, and increase in fetal systemic blood flow pressure), US examination conditions can be very limited [26], [27]. As fetal CMR continues to overcome previous limitations by implementing feasible gating techniques and motion compensation methods, it is now able to bridge this gap. However, insufficient image quality caused by spontaneous fetal movements remains a major challenge of the technique. In this study, 3% of CMR exams must be excluded from further analysis due to significant artifacts caused by arbitrary fetal motion and 97% of ventricles could be successfully assessed, resulting in an overall success rate of approximately 94%. This is widely consistent with previous findings on fetal CMR FT, which reported success rates of 88% and 100% [14], [15]. Compared to other fetal CMR techniques, e.g., it surpasses success rates of 75% in four-dimensional flow CMR and 83% in cine CMR of the aortic isthmus [14], [15], [28], [29]. The remarkable feasibility is likely attributed to its reliance on a single four-chamber cine slice without necessitating dedicated sequences. This advantage renders it highly appealing for evaluating cardiac function in the fetus, particularly in challenging examination conditions.

The eight normal control fetuses from this study had a mean LV GLS of −18.6%, LV GRS of 25.7%, LV SR of −1.6 s^−1^, and RV GLS of −16.1% at a mean gestational age of 249 days (i.e., 35 weeks + 4 days). This aligns with the latest longitudinal cohort study using STE in 124 healthy fetuses, which reported an LV GLS of −19.64%, LV SR of −1.6 s^−1^, and RV GLS of −17.31%. Furthermore, the findings from this study are in line with previous both longitudinal and cross-sectional cohort studies using speckle tracking as well as with the initial CMR FT results indicating normal values of −19% and −18.9% for LV GLS, respectively, −1.6 s^−1^ for LV SR, and −16.2% for RV GLS [14], [15], [30], [31], [32], [33]. Nevertheless, it is important to acknowledge the considerable variability of strain values depending on techniques and analysis approaches. For instance, echocardiographic measurements ranged from −12.3% to −21.1% for LV GLS and −12.8% to −19.5% for RV GLS, all within a comparable gestational age of 36–38 weeks [30], [34]. These conflicting results may be attributed to differences in study design (such as longitudinal vs cross-sectional), the quality of acquired four-chamber views, and differences in US devices or speckle tracking algorithms [25]. Likewise, variations in strain measurements have been noted between studies investigating CMR FT in adults, likely attributable to differences in sex and age, with GRS being less consistent than GLS [35]. Hence, the encouraging agreement observed in initial fetal strain values derived from CMR FT warrants validation through further studies involving larger cohorts of fetuses at varying gestational ages.

In contrast to the results reported by Dargahpour Barough et al. [15], our study did not observe statistically significant variances in LV and RV strain parameters between normal control fetuses and fetuses diagnosed with CHD. Within the CHD group, however, a wider range of strain values was observed compared to controls. While fetuses with dTGA and TOF exhibited a significant overlap with normal values, fetuses with HLHS and RV-dominant AVSD demonstrated poorer strain performance. Conversely, fetuses with CoA exhibited even exaggerated performance compared to normal controls. Therefore, the disparity in findings is likely attributable to the heterogeneity within the CHD cohort rather than methodological discrepancies between the two studies.

Among the different CHD subgroups, significant impairment of myocardial strain was evident in fetuses with a higher likelihood of a univentricular outcome; consequently, severe reductions in LV GLS were observed in fetuses diagnosed with HLHS and RV-dominant AVSD. Furthermore, diminished LV GRS was notable in HLHS cases, while compromised RV GLS was apparent in fetuses diagnosed with critical PS/PA. In line with findings from previous studies using STE, strain performance of the dominant ventricle in these fetuses (specifically, RV in HLHS and AVSD vs LV in PS/PA) was comparable to normal controls [24]. This is of particular importance for fetuses likely to undergo a univentricular surgical approach, given that the performance of the single ventricle exerts a substantial influence on clinical prognosis [24]. Moreover, e.g. in HLHS, the interventricular interdependence of LV morphology and loading conditions and RV performance can be individually assessed [9], [36]. Thus, fetal strain analysis could contribute to relevant physiological information when considering univentricular versus biventricular outcome.

A close association between ventricular conditions and strain values can also be assumed from the findings in the CoA subgroup, where the increase in strain performance may indicate adequate myocardial adaptation to the increased afterload. This finding is consistent with earlier reports using STE, where fetuses with increased LV afterload pressure, such as those with aortic valve stenosis and CoA, exhibited higher LV strain values compared to fetuses with other CHD and normal fetuses [8]. Similarly, in serial measurements using speckle tracking, fetuses with aortic stenosis and CoA demonstrated an increase in LV strain, suggesting an appropriate response to pressure loading [24]. In contrast, other studies reported decreased LV strain in fetuses with CoA compared to normal fetuses, attributing this to increased LV and aortic wall stiffness and shortened coronary perfusion time [37], [38]. Therefore, it still remains unclear whether these findings genuinely reflect physiological adaptations during late pregnancy and might even provide prognostic insights to differentiate between CoA requiring postnatal treatment and mere arch hypoplasia. This issue needs further clarification through future outcome studies involving a larger cohort of compromised fetuses.

Previous echocardiographic assessment in fetuses with TOF showed impaired longitudinal strain in both ventricles with even greater impairment of the LV. This may be attributed to increased LV preload through the ventricular septal defect and narrowed LV outflow size due to the aorta straddling the interventricular septum [39]. However, only in 48% of all fetuses with TOF abnormal longitudinal strain measurements were found [39]. This agrees with the results of this study, where no significant differences in RV and LV strain were observed between compromised fetuses and normal controls. Likewise, no significant differences were found between fetuses with dTGA and controls. This corresponds with previous results from STE in fetuses with dTGA requiring urgent postnatal atrial septostomy, whereas those without foramen ovale restriction had abnormal global contractility measurements [40]. As foraminal flow restriction is considered primarily a third-trimester phenomenon [40], the prognostic value of CMR FT in late third-trimester pregnancy should be further evaluated to identify fetuses with dTGA that require septostomy.

### 4.1 Limitations

General limitations of CMR FT include its dependence on adequate temporal resolution, particularly at high fetal heart rates. In this study, the average temporal resolution was 17.5 ms, resulting in an average frame rate of 57 frames per second with 25 reconstructed phases per cardiac cycle. This frame rate is considerably higher compared to those reported in previous studies on fetal CMR FT, which reported frame rates of 46 and 27–55 frames per second, respectively [14], [15]. However, it is lower than the frame rates of STE and may result in an underestimation of peak strain values due to missed short-lived events during the isovolumic period [41], [42]. This should be considered when interpreting myocardial strain in a clinical context, e.g., by comparison with normal values derived from CMR FT. Furthermore, fetal motion can significantly affect the accuracy of strain measurements. Moreover, the lack of consensus on different measurement techniques and software packages may restrict the generalizability of study results. Specific limitations of the current study are the lack of direct comparison to echocardiographic data due to the retrospective study design. The software used in this study did not support analysis of regional strain, which could have implications for the interpretation of study findings (e.g., assessing interventricular interdependence by septal wall analysis). Additionally, despite a considerable total number of fetuses, the quantity within each CHD subgroup was relatively low. Finally, as myocardial strain is expected to change during the course of pregnancy, the values reported in this study may specifically apply to fetuses in the advanced third trimester.

### 4.2 Conclusions

CMR FT using DUS-gated cine imaging is a feasible approach for myocardial strain assessment in fetuses with different forms of CHD and is highly reproducible. Depending on the underlying heart defect, distinct patterns of strain performance are evident across different CHD subgroups with considerable overlap with normal values. The potential prognostic value of biventricular strain assessment in different clinical scenarios, including the identification of fetuses requiring urgent postnatal intervention, warrants further research.

## Funding

None.

## Author contributions

**Ulrike Attenberger:** Writing – review and editing, Resources. **Annegret Geipel:** Writing – review and editing, Project administration, Methodology. **Brigitte Strizek:** Writing – review and editing, Project administration, Methodology. **Julian Luetkens:** Writing – review and editing, Supervision, Project administration, Methodology, Conceptualization. **Thomas M. Vollbrecht:** Writing – original draft, Visualization, Validation, Methodology, Investigation, Formal analysis, Data curation, Conceptualization. **Christopher Hart:** Writing – review and editing, Project administration, Investigation, Conceptualization. **Christoph Katemann:** Writing – review and editing, Software, Resources, Methodology. **Alexander Isaak:** Writing – review and editing. **Claus C. Pieper:** Writing – review and editing. **Daniel Kütting:** Writing – review and editing.

## Declaration of competing interests

The authors declare that they have no known competing financial interests or personal relationships that could have appeared to influence the work reported in this paper.
